# Preparation of Environmentally Friendly Glueless Boxwood Timber by Acidic Environmental Treatment and High-Temperature Pressing

**DOI:** 10.3390/polym15010011

**Published:** 2022-12-20

**Authors:** Hongfei Huo, Feifei Song, Yang Yang, Lei Zhang, Xu Zhang, Jijuan Zhang, Kong Yue, Zhongfeng Zhang

**Affiliations:** 1 College of Furniture and Art Design, Central South University of Forestry and Technology, Changsha 410004, China; 2 Green Furniture Engineering Technology Research Center of National Forestry and Grassland Administration, Changsha 410004, China; 3 Green Home Engineering Technology Research Center in Hunan, Changsha 410004, China; 4 Dongyang Furniture Research Institute, Dongyang 322100, China; 5 School of Civil Engineering, Nanjing University of Technology, Nanjing 210000, China

**Keywords:** glue-free gluing, acid condensation, environmentally friendly wood-based panels, boxwood

## Abstract

In the context of high-quality development, environmental issues are being paid more and more attention to, and the release of free formaldehyde has become a major problem that needs to be solved. Glueless plywood mainly adopts natural substances as raw materials, without adding chemical products, such as resin adhesives, and it does not contain harmful substances, such as formaldehyde. Glueless plywood is a green product that causes no pollution in the environment and no harm to the human body. In this study, the corresponding weak-phase components in boxwood were pre-delivered by an acidic environmental treatment, and the high-temperature and high-pressure compacting process produced a glueless boxwood panel with excellent water resistance and mechanical properties, while remaining environmentally friendly.

## 1. Introduction

With the improvement of people’s living standards and environmental awareness, the demand for eliminating the harm caused by formaldehyde gas has become increasingly strong, and the problem of excessive formaldehyde release from man-made panels has become a hotspot of concern. In the production process of three major traditional wood-based panels (fiberboard, particleboard, plywood), the large amount of formaldehyde adhesives are seriously harmful to people’s health. Most traditional wood adhesives are synthetic resin adhesives, containing a large amount of free formaldehyde, which not only causes air pollution, but also poses a great danger to people’s health, so glue-free panels are of great concern. The search for glueless wood-based raw materials and the development of green, environmentally friendly wood-based panels has become a hot Issue in the current wood industry [1].

Glueless bonding is a technology that does not require the addition of traditional synthetic resin adhesives but relies primarily on the chemical composition of the material itself to achieve “self-adhesive” bonding under specific process conditions. The principle is to activate the surface of plant fibers through a special treatment and, at the same time, produce an adhesive-like substance that allows the glued material to be glued into sheets under hot-pressing conditions. Because the bonding phenomenon occurs on the surface of the material, the bonding effect of the adhesive is influenced by the state and nature of the surface of the bonded material, and the bonding effect of the glue-free laminate is also influenced by the nature of the surface of the wood fiber. Glueless plywood mainly uses natural substances as raw materials, and rarely adds chemical products such as resin adhesives. Additionally, glueless plywood does not contain formaldehyde and other harmful substances and it is a green product that causes no pollution in the environment and no harm to the human body, so it is suitable for manufacturing bedroom furniture, children’s furniture and other furniture with high requirements for environmental protection [2]. Compared to synthetic resin, non-glued plywood is widely used because of its excellent properties, such as environmental protection, non-toxic, anti-corrosion and heat insulation. The corrosion resistance of glue-free plywood is significantly higher than that of synthetic resin plywood, and it is suitable for use in environments with strict requirements for corrosion and insect protection. Compared with air-dried wood of a similar density, the thermal conductivity of glue-free plywood is small, and the thermal conductivity is low, so it has an excellent heat-insulation performance and can be used for floor matting, interior wall decorative panels. Additionally, other heat-insulation-manufacturing processes do not use additional adhesives, so the glue-making and mixing processes are eliminated in the manufacture of chipboard. Furthermore, there is no glue pre-curing problem, and the sanding process can be eliminated. This not only simplifies the production process, but also saves labor and material resources. The mass production of glue-free plywood can not only alleviate the contradiction between the supply and demand of wood resources, reduce the consumption of forest resources and protect the human ecological environment, but also solve the free formaldehyde pollution of indoor decoration and furniture, which is in line with the current policy of creating a resource-saving and environmentally friendly society advocated by the state, as well as having good ecological and social benefits [3].

The poor wet strength of glueless plywood on the market and the large swelling rate of its water absorption thickness are attributed to the insufficient activation of the wood fiber surface; therefore, how to activate the wood fiber surface to release as many active hydroxyl groups as possible has become the focus of research. Common pretreatment methods for wood fiber raw materials include oxidation bonding, enzyme activation, alkali solution activation, acid-catalyzed polycondensation, natural material conversion, etc. Acid has a strong destructive effect on wood. The use of acid can degrade cellulose and hemicellulose in wood, its degradation products dehydrate and generate furfural substances and this material can be produced under hot-pressing conditions of the polymer which can play the role of an adhesive so that the wood is glued [4]. The size of the crystalline zone of cellulose, the degree of crystallinity and the orientation of microfibrils in each wall layer of the cell have a strong influence on the elasticity of the cell wall. The rigidity of the wood cell wall is mainly determined by cellulose. Hemicellulose and lignin can also increase the rigidity of the cell wall, but their contribution is lower than that of cellulose. In contrast, the viscosity of the wood cell wall is mainly determined by both lignin and hemicellulose, especially in the lateral properties of the cell wall. Differences in the content, type or nature of the two can cause differences in the viscoelasticity of the wood cell wall. Lignin is weakly hygroscopic and little affected by moisture, and as the degree of delignification increases, the hygroscopic capacity of wood increases and the mechanical hygroscopic creep increases significantly. Hemicellulose is highly hygroscopic because it contains more hydrophilic groups, and the partial removal of xylan and glucomannan has no significant effect on the mechanical hygroscopic creep of single wood fibers. However, the complete removal of hemicellulose significantly reduces the mechanical hygroscopic creep of single wood fibers [5]. Hemicellulose in wood usually consists of several types of sugar units and contains acetyl and methyl substituents. In its natural state, hemicellulose is amorphous, has a low degree of polymerization and is highly reactive [6]. In acidic media, the glycosidic bonds of hemicelluloses are susceptible to opening, which allows them to undergo degradation. Compared to cellulose and lignin, hemicelluloses in wood are the least thermally stable. During the heat treatment of wood, hemicellulose is firstly deacetylated by heat, and the acetic acid that forms can act as an acidic catalyst to accelerate the dehydration of hemicellulose, remove hydrophilic hydroxyl groups and carbonyl groups in hemicellulose, and promote the depolymerization of polysaccharides to form furfural and hydroxymethyl furfural [7,8]. Additionally, these products can undergo dehydration, polymerization, condensation and isomerization of polyhydroxy compounds under high-temperature and pressure conditions [9,10,11].

**Figure 1:**
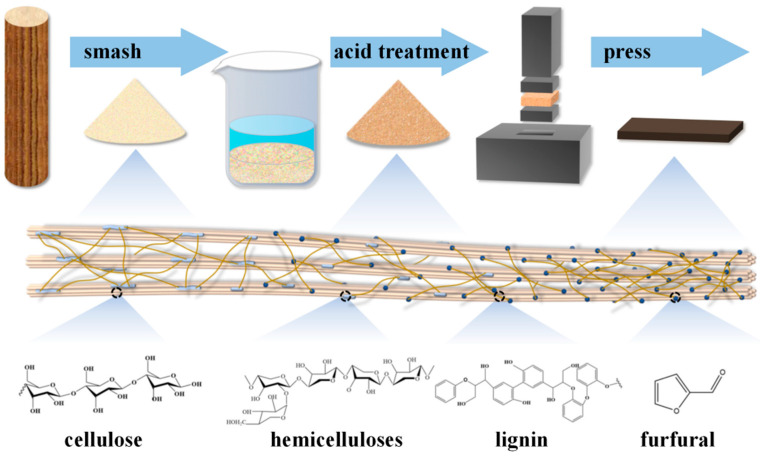
Preparation process of environmentally friendly glueless boxwood panels.

Boxwood is a common wood used in valuable furniture and carving artworks, but it is slow-growing and small in diameter, making it difficult to achieve high-value utilization in production and processing. As shown in Figure 1, this experiment was carried out using an acidic environment treatment to remove the corresponding weak-phase component hemicellulose from boxwood under the action of external factors, while using the masking or substitution effect of the reaction medium to reduce the hydrophilic hydroxyl group of the reactive group in the wood component. This was followed by compression compacting, which makes the wood’s own cellular tissue arrangement more compact with the help of thermo-mechanical force, so that the internal stress release of the compressed wood, the internal molecular, the formation of molecular bridges and water-repellent bonds within the compressed wood improves the compactness of the cellular tissue.

## 2. Materials and Methods

Experimental procedure: The peeled boxwood without defects was selected. A 60–80 mesh powder was obtained using a high-speed crusher for the experiment. Acetic acid was obtained from Fuchen Chemical Reagent Co., Ltd TianjinChina, and the purity was analytically pure. The boxwood powder was put into a solution with an acetic acid concentration of 6%, and the heating temperature of the oil bath (HH-6J, Lang Yue Instrument Manufacturing Co., Changzhou, China ) was set to 150 °C. The stirring time was 5 h. Then, the solution was cooled to room temperature. Subsequently, the mixture was poured out, filtered, washed with double-distilled water and dried at 60 ± 3 °C. The moisture content of the raw wood powder (RWP) and dealt wood (DW) were controlled at 6% and 8%, respectively. For hot pressing, 8 g of powder was placed into the mold, the pressure was set to 75 MPa, the temperature was set to 180 °C and the time was set to 90 min.

The test method was carried out according to GB/T17657-2013 “Physical and chemical properties of man-made boards and veneered man-made boards test method” to detect its physical and chemical property indexes, such as brightness, density, water-absorption thickness-expansion rate, flexural strength, elastic modulus, tensile strength, etc. A UV-Vis spectrophotometer (UV-Vis, UV-2600, Shimadzu, Tokyo, Japan) was used to determine the UV-Vis spectra, and the spectral scan range was 200~800 nm with a 1 nm increment and a fast speed. A contact angle meter (DSA 100, Krüss, Germany) was used to test the contact angle of the reference liquid on the sheet surface by the static droplet method. The specimens were measured using a Fourier-transform infrared spectrometer (FTIR, Nicolet iS10, Thermo Scientific, New York, NY, USA) in the wave number range 4000–400 cm^−1^ with a resolution of 4 cm^−1^ and with 64 scans. The specimens were analyzed by thermogravimetric/differential thermogravimetric analysis (TG/DTG) using a DTG-60 thermal analyzer (Shimadzu, Tokyo, Japan) with a ramp-up rate of 10 °C/min to 700 °C. The samples were tested for microscopic morphology using an FEI Quanta 200 scanning electron microscope (New York, NY, USA) device with an accelerating voltage of 20 kV, and all samples were gold sprayed prior to testing. The crystalline properties were characterized using an X-ray diffractometer (XRD), with 200 mesh samples taken by grinding and sieving with a Cu target, Ka radiation λ = 0.154 nm, voltage of 40 kV, current of 35 mA, scan speed of 5°/min, step size of 0.05°, and a scan range of (2θ 5°~ 50°). XPS was performed using an X-ray photoelectron spectrometer (XPS, ESCALAB 250Xi, Thermo Scientific, New York, NY, USA) with an aluminum target X-ray source (Al Kα, hv = 1486.6 eV), a beam spot of 400 μm, charge correction using contaminated carbon C1s = 284.8 eV for correction, a narrow spectrum scan of 50 eV for flux energy, a full spectrum scan at 100 eV and a vacuum of 2 × 10^−7^ mbar.

## 3. Results and Discussion

### 3.1. Performance Analysis

**Figure 2:**
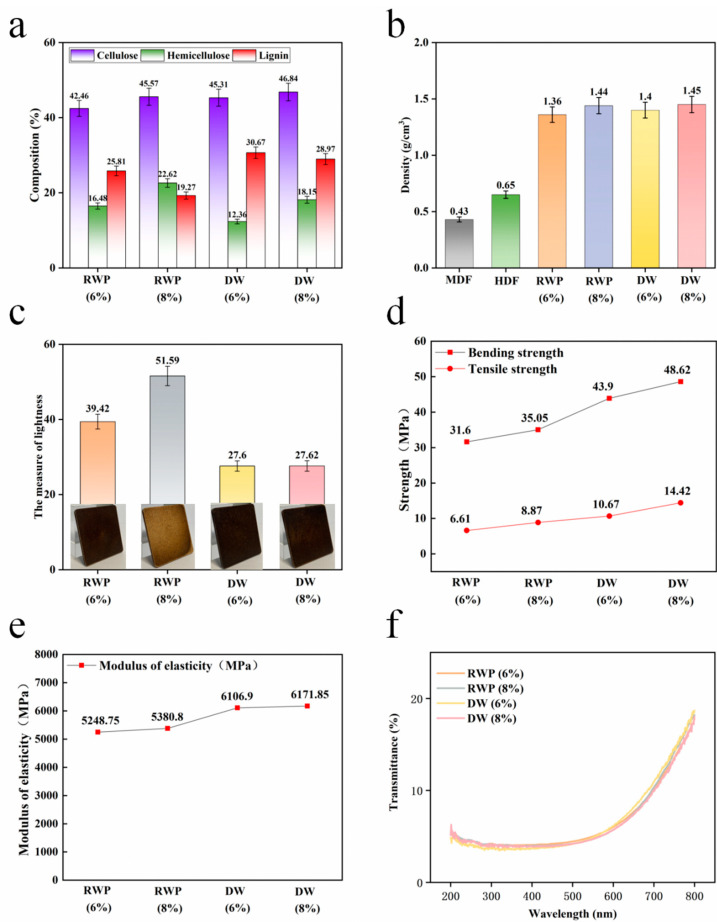
(**a**) Comparison of lignin, cellulose and hemicellulose content; (**b**) comparison of density; (**c**) comparison of brightness; (**d**) comparison of flexural strength and compressive strength; (**e**) comparison of modulus of elasticity; (**f**) comparison of UV transmittance.

As shown in Figure 2a, in the composition measurement, it was found that the percentage of cellulose and lignin in the DW became higher and the percentage of hemicellulose decreased significantly. This is due to the degradation of hemicellulose and a small portion of cellulose during the acid treatment, as well as hot pressing, while lignin keeps the main structure stable. As shown in Figure 2b, under the effect of hot pressing, the fibers in RWP are closely bonded together, and the density of RWP boards with a 6% and 8% moisture content reaches 1.36g/cm^3^ and 1.44 g/cm^3^, respectively, while the density of DW boards with a 6% and 8% moisture content is a little higher, reaching 1.40 g/cm^3^ and 1.45 g/cm^3^, respectively, indicating that the fibers are more closely bonded after acid treatment.

As shown in Figure 2c, in the brightness test, the original color of RWP was retained to a large extent, while the DW produced a dark, high hardness layer on the surface due to denser bonding. Meanwhile, in Figure 2f, which shows the UV transmittance test, it can be seen that DW lumber had lower transmittance in the visible wavelength range of 380–800 nm compared to RWP lumber, indicating that its internal structure was denser and more thoroughly bonded.

As shown in Figure 2d,e, the static flexural strength of the RWP board reached 31.6 MPa and 35.05 MPa, 163.33% and 192.08% higher than particle board. In addition, the static flexural strength of the DW board reached 43.9 MPa and 48.62 MPa, 265.83% and 305.17% higher than particle board, which reached 18.65%, and the static flexural strength of the DW board increased by 38.92% and 38.72% at the same moisture content. The modulus of elasticity of the RWP board reached 5248.75 MPa and 5380.8 MPa, 176.25% and 183.2% higher than particle board, and 38.13% and 41.6% higher than the HDF(High Density Fiberboard). The modulus of elasticity of the DW board reached 6106.9 MPa and 6171.85 Mpa, 221.42% and 224.83% higher than the particle board, and 224.83% higher than the HDF. The results are 224.83%, 60.71% and 62.42% higher than that of the high-density fiberboard, and the modulus of elasticity of DW the boards increased by 16.35% and 14.70% when the moisture content was the same. The tensile strength of the RWP board reached 6.61 Mpa and 8.87 Mpa, while the tensile strength of the DW board reached 10.67 MPa and 14.42 MPa, and the tensile strength of the DW board increased by 61.42% and 62.57% with the same moisture content. Its mechanical properties meet the needs for furniture.

**Figure 3:**
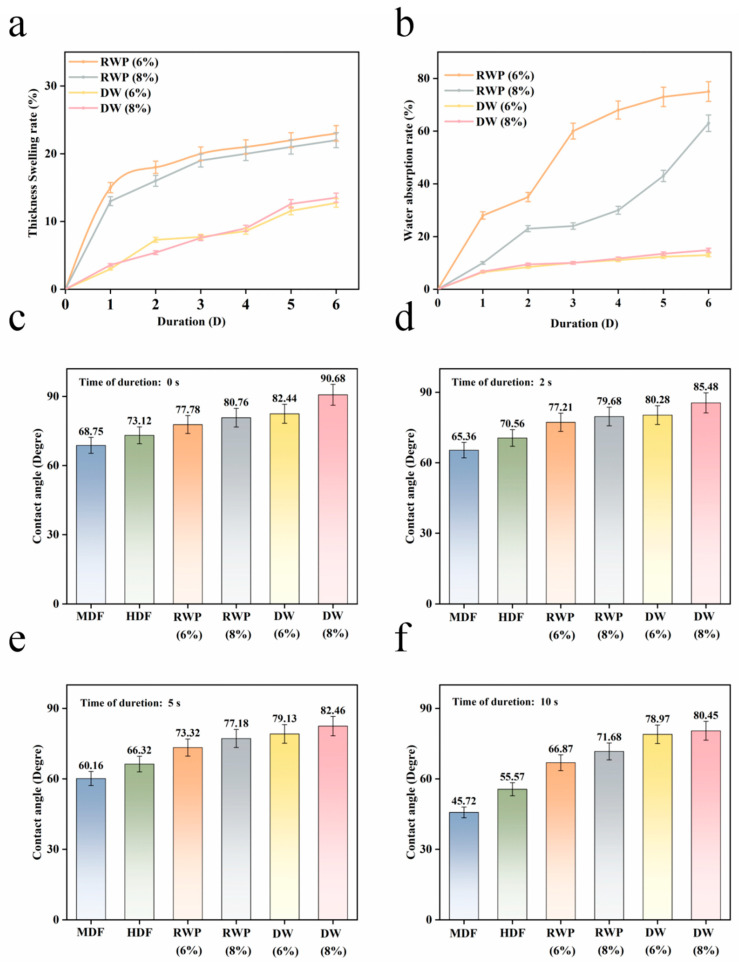
(**a**) Comparison of water-absorption thickness-swelling rate; (**b**) Comparison of water absorption rate; (**c**–**f**) Comparison of contact angle (0 s, 2 s, 5 s, 10 s).

As shown in Figure 3c–f, in the 0S contact angle test, the RWP board and the DW board both reached more than 75 degrees. In the 10s contact angle test, the contact angle of the RWP board and the DW board only decreased by 14.03%, 12.24%, 4.21% and 11.28%, compared to MDF (Middle Density Fiberboard), which decreased by 34.20%. Additionally, the high-density fiber board decreased by 24.01%, significantly improving hydrophobicity. After acid treatment, the contact angles of the boards were improved. The initial contact angle on the surface of the boxwood boards increased with the increase in moisture content. Wood is a hydrophilic material because of the large number of hydroxyl groups on the microscopic surface of the wood, and because of the reduction in hydroxyl groups due to the formation of hydrogen bonds during treatment [12]. As shown in Figure 3a,b, in a six-day water-absorption thickness-swelling test, the thickness of the DW board swelled by only 3% on day 1, compared to about 14% for the RWP board, which greatly reduced its water absorption. After six days, the thickness of the DW board swelled by only 11%, compared to about 22% for the RWP board, which greatly reduced its water absorption.

**Figure 4:**
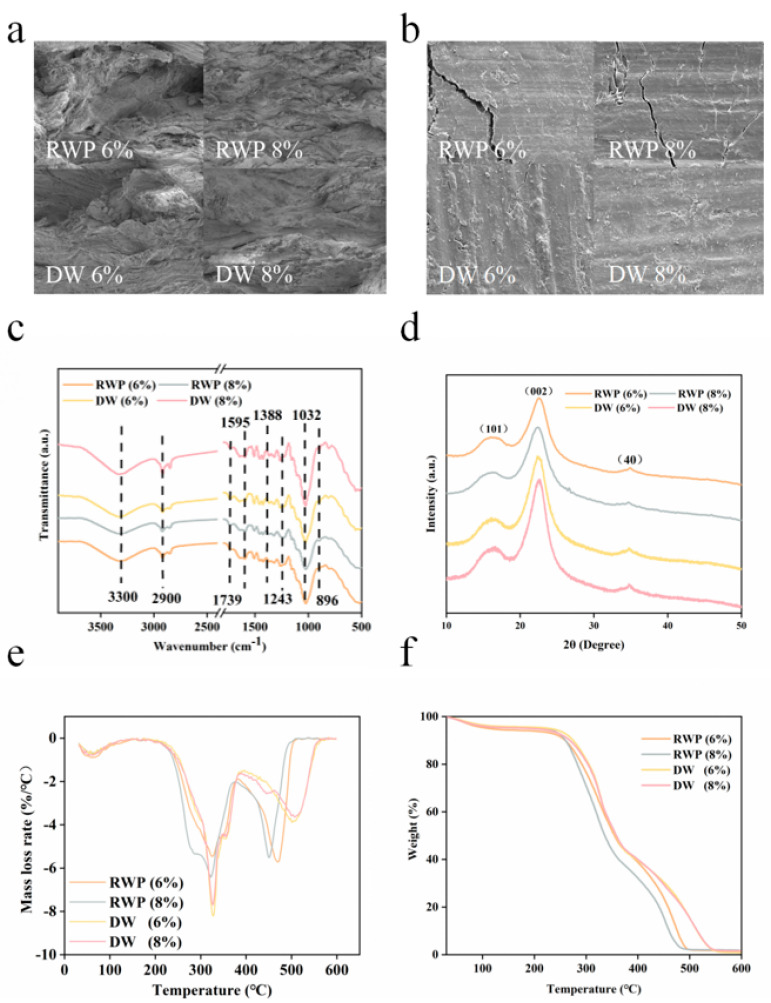
(**a**) Comparison of cross−sectional electron micrographs; (**b**) Comparison of surface electron micrographs; (**c**) Comparison of FTIR; (**d**) Comparison of XRD; (**e**) Comparison of weight-loss rate; (**f**) Comparison of thermogravimetric curves.

Compared to the RWP board, the improvement in hydrophobicity and static-bending strength of the DW board and the improvement in its fiber bonding degree had a great relationship, which we can verify through an electron microscope test. As shown in Figure 4a,b, the fiber bundle interweaving was not obvious enough on the cross-section of RWP board: the gap between fiber and fiber was larger, there were more cavities, which can be distinguished clearly, and the bonding between fibers was weaker, which shows small bonding strength. With the increase in moisture content, the fiber form was more complete, there were gaps between the fibers, the fibers were bonded together to produce a bonding effect, the bonding between the fibers was enhanced, making the generated fiber bundles interweave with each other, and the bonding strength increased. On the surface of the board, surface defects, such as cracking, were reduced and are flatter and denser due to the closer bonding of the wood flour fibers after acid treatment [13].

### 3.2. Mechanistic Analysis

As shown in Figure 4c, from the FTIR results, it can be seen that the absorption peak at 3300 cm^−1^ was a hydroxyl stretching vibration with reduced absorption intensity, shifted to the lower wave number direction, which indicates that the hydroxyl groups formed hydrogen bonds after acid treatment. The absorption peak at 2900 cm^−1^ was caused by a methylene C–H stretching vibration, which was weakened after acid treatment and shifted to the lower wave number direction. The absorption peak at 1739 cm^−1^ was characterized by carbonyl groups and C=O stretching vibration in esters with increased intensity. The characteristic absorption peak at 1595 cm^−1^ characterized the aromatic ring of lignin, which was not significantly damaged after acetic acid treatment. The intensity of the 1243 cm^−1^ C-O stretching vibration of lignin remained unchanged. The absorption intensity of the 1032 cm^−1^ C–O–C stretching vibration decreased. The absorption peak at 896 cm^−1^ is that of the glycosidic bond in cellulose, and the absorption intensity was reduced, indicating that part of the cellulose was degraded, breaking the glycosidic bond [14]. From the above, it is clear that hemicellulose and cellulose are degraded after exogenous acid treatment, which has essentially no effect on lignin. Part of the hemicellulose dissolves in aqueous solutions, and part of the hemicellulose is degraded to small molecular sugars by the action of acid; thus, the structure and composition of the remaining hemicellulose is greatly altered after exogenous acid treatment. As the water content increases, the number of hydroxyl groups decreases and moves toward lower wave numbers, indicating that the hydroxyl groups interact to form hydrogen bonds, which increases the bonding force between fibers and facilitates fiber bonding into boards [15,16]. At the same time, the glycosidic bond is broken to produce furfural, which later undergoes a dehydrogenation and polycondensation reaction under a high temperature and pressure to produce part of the furan ring and furan resin, which provides bonding and water resistance [17,18].

As shown in Figure 4d, during the XRD analysis, the (101) plane diffraction peak and the (002) plane diffraction peak were the peaks that mainly affected the crystallinity of the wood. The (101) face diffraction peak was extremely small and the diffraction intensity of 2θ = 18° was the scattering intensity of the diffraction from the amorphous region in the wood fiber. The (002) crystalline surface diffraction peak was of an extremely large value, and the diffraction intensity of the wood fiber was 2θ = 22°. The diffraction peaks of DW became stronger after acid treatment because the acid broke the envelope of lignin and hemicellulose to cellulose, hemicellulose was hydrolyzed by acid, and the surface area of cellulose increased. The diffraction peaks at other positions were not very relevant to the crystallinity calculation. Crystallinity was calculated according to the equation of crystallinity by the Segal method. The crystallinity of RWP lumber and DW lumber with a 6% and 8% moisture content were 57.62%, 66.66%, 85.04% and 83.03%, respectively. The crystallinity increased significantly after chemical treatment. This is because wood has crystalline and non-crystalline zones, and the water molecules contained in wood are usually in the free state in the non-crystalline zone, and the mass of the non-crystalline zone gradually decreases with the loss of water molecules. In the wood structure, hemicellulose itself also has adhesive forces, and acidic solvents dissolve the connecting bonds between hemicellulose molecules, so that hemicellulose and its degradation products are connected with cellulose and lignin, and the forces between multiple components are greater [19,20].

As shown in Figure 4e,f, in the analysis of the thermogravimetric results, the first stage of wood pyrolysis is the dehydration stage, which occurs between room temperature and near 110 °C. This is mainly due to the process of vaporization of water contained in wood. The weight loss resulting from this stage is reduced after acid treatment because the moisture content of acid-treated wood is less than that of untreated wood. The second stage is the rapid pyrolysis stage, which occurs at 250–390 °C and is mainly due to the decomposition of the wood chemistry, with the thermal weight loss occurring near 345 °C, mainly arising from the degradation of lignin. The third stage is the slow pyrolysis stage, i.e., the charring stage, which mainly involves the degradation of the remaining lignin and produces little gas, and the curve tends to level off. It can be seen from the figure that the thermal stability of the acid-treated samples is better than that of the untreated samples, which is due to the fact that acid treatment causes lignin to condense and increases internal hydrogen bonding [21,22].

**Figure 5:**
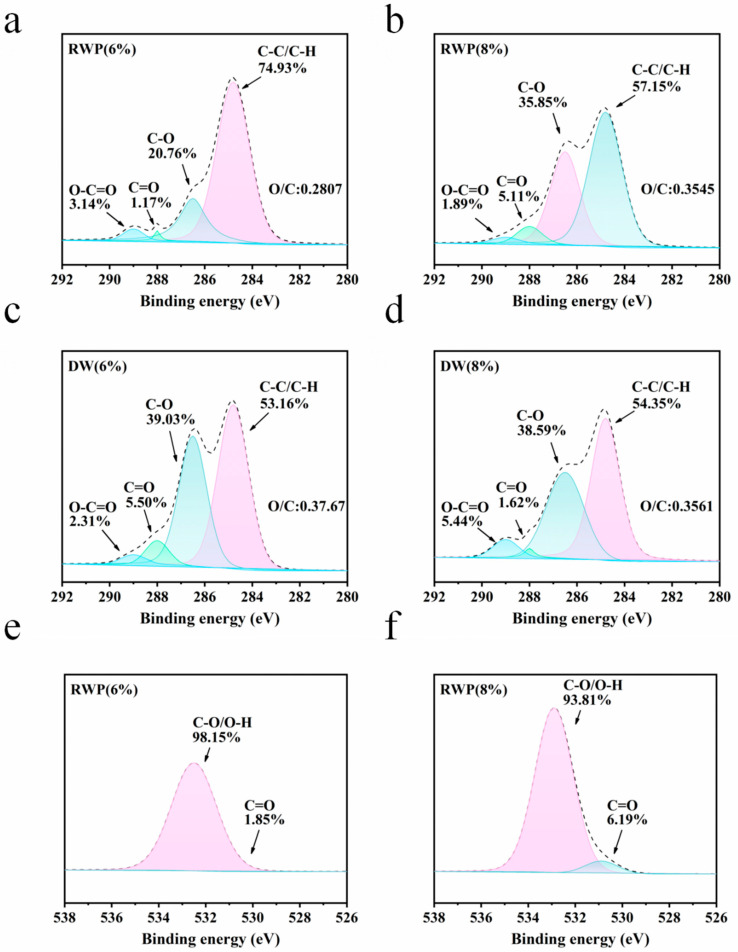
(**a**–**d**) XPS carbon fractionation results analysis; (**e**,**f**) XPS oxygen fractionation results analysis.

**Figure 6:**
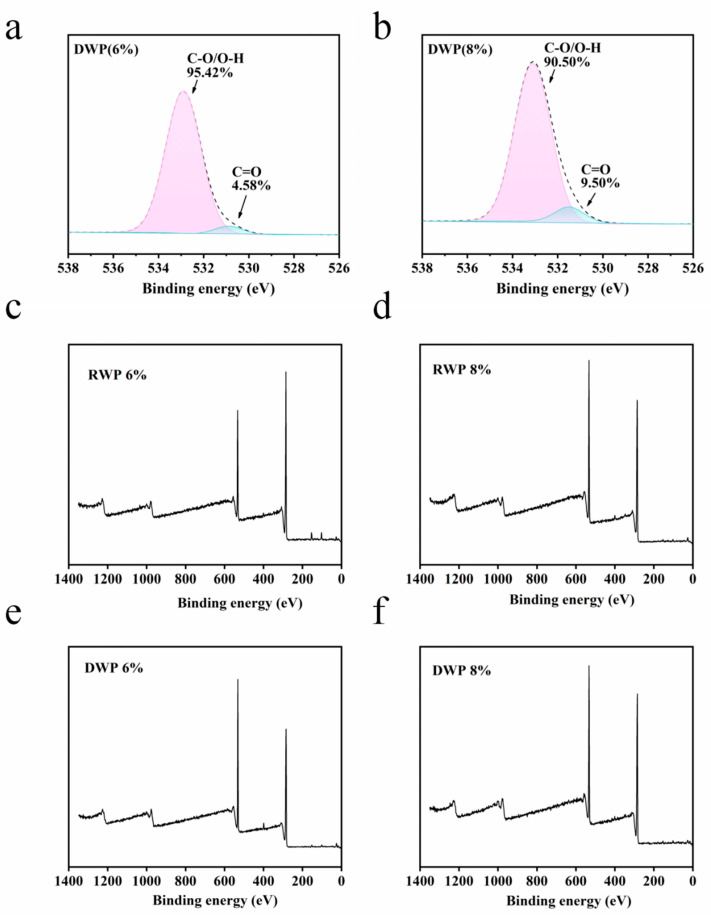
(**a,b**) XPS oxygen fractionation results analysis; (**c**–**f**) XPS carbon peak results analysis.

In the analysis of the XPS results, C1 represents C atoms attached only to other saturated C or H atoms (–C–C– or –C–H, 284.8 eV), mainly from lignin, fatty acid and wax extracts in wood; meanwhile, C2 represents C atoms attached to only one non-carbonyl O atom (–C–O–, 286.5 eV), mainly from cellulose and hemicellulose in wood, fatty acids, waxes and other extractives; C2 represents C atoms attached to only one non-carbonyl O atom (–C–O–, 286.5 eV), mainly from the hydroxyl groups in cellulose and hemicellulose in wood; C3 represents C atoms attached to one carbonyl-like O atom or two non-carbonyl-like O atom (–O–C–O– or –C==O, 288 eV), mainly from the carbonyl groups of lignin and hemicellulose in wood; C4 represents the C atom attached to a carbonyl O atom and a non-carbonyl O atom (–O–C–O– or –C==O, 288 eV); and C4 represents the C atom attached to a carbonyl O atom and a non-carbonyl O atom C atom (–O–C==O, 289 eV). O1 represents an O atom connected to a C atom by a double bond (C==O, 530 eV), and O2 represents an O atom connected to two C or H atoms only by a single bond (C–O, 532 eV). The relative oxygen to carbon ratio can better reflect the surface properties of wood fibers, as shown in Figure 5 and Figure 6. The oxygen to carbon ratio (O/C) increases to different degrees after acid treatment, indicating an increase in the number of hydrophilic groups on the surface. The relative value of C1 decreases and the relative value of C2 increases. On the one hand, this is because the acid treatment reduces the extractive content in the wood chips, and on the other hand, it is because lignin has a higher thermal stability compared to cellulose and hemicellulose, as well as because of the treatment. The relative content of lignin on the wood surface increases and the content of polysaccharides (cellulose and hemicellulose) decreases after treatment [23]. The decrease in the O1 peak area and the increase in the O2 peak area of DW compared to the RWP fraction indicate that the percentage of oxygen atoms with high binding energy on the surface of wood fibers increases after acid treatment [24].

## 4. Conclusions

After acetic acid pretreatment, some cellulose and hemicellulose in boxwood powder are degraded and some lignin is leached out, but the benzene ring structure is not destroyed, and the structural integrity is maintained. The new stable polymer produced during the hot-pressing process produces an adhesive effect, while the hydroxyl radicals combine with each other to form hydrogen bonds. Furfural produces part of the furan ring and furan resin, which provide the bonding properties that help to form the panels. Without adding any adhesive, the performance of boxwood lumber can meet the needs of furniture and truly meet the requirements of glueless environmental protection. At the same time, the moisture content of boxwood powder has a greater impact on the strength of the hydrogen bonding of the board. With a moisture content of 8%, the board is more closely combined, leading to a better performance.

## Data Availability

Data is contained within the manuscript.
